# 

**Published:** 1917-02-17

**Authors:** 


### CONTENTS.

| | PAGE |
|---|---|
| **The Mobilisation of the Medical Profession** ... | 395 |
| **Money and Trusteeship** ... ... ... ... ... | 396 |
| **Hospital and Institutional News** ... ... ... | 397 |
| The New Year's Honours—The Orders of Knighthood—An Alteration of the Laws of the Middlesex Hospital—Proposed Laboratory for North Wales—Lord Devonport and the Hospitals—Munificent Bequests to the Sunday Fund—The Brentford Infirmary Dispute—Another Benefaction for the Welsh Medical School—A Big Extension in Ireland—A Red Cross Herring—The Liability of a Husband for Medical Attendance on His Wife—A Local Medical Directory—A Hospital Treasurer on Votes for Women—The Difficulties of Food Restriction—A Hospital's Medical Staff—Women as Presidents of Cottage Hospitals—Abbreviated Annual Reports—A Crisis at Chorlton-on-Medlock—Military Hospital Expansion at Newport—Wounded Allies' Relief Committee—A Much-Endowed War Hospital—Queenly Tribute to Nurses—The Risk of Libel in Medical Practice—The Discoveries of Convalescence—Meatless Day at the British Home, Streatham—The Career of the Workhouse Child—A New Editor at Birmingham—The "Craigleith Chronicle"—A Feast of Illustrations—This Week's Drug Market—To Our Readers. | |
| **Naval Medicine in the Great War** ... ... | 403 |
| **Cats as Carriers of Disease** ... ... ... | 405 |
| Inflammation of the Eye from Infected Fur. | |
| **Military Hospitals in India** ... ... ... | 406 |
| By an Ex-Patient. | |
| **The Russian Union of Zemstvos** ... ... ... | 407 |
| A Fine Organisation for the Care of the Wounded. | |
| **From Across the Seas** ... ... ... ... | 408 |
| **Hospital Results** ... ... ... ... ... | 408 |
| **The Matrons' and Sisters' Department** ... | 409 |
| The Royal British College of Nursing. The Matrons and the College. Round the Hospitals. An Opportunity Not to be Missed—Sowing Disunion and Reaping Destruction—The Chairwoman of the Union Executive—The New Matron of St. Mary's—Belfast and the College—The Central Committee's Policy Dead. | |
| **A School for Massage** ... ... ... ... | 411 |
| Developments at Guy's Hospital. | |
| **Matrons' and Sisters' Appointments** ... ... | iv |
| **Personalia**... ... ... ... ... ... ... | iv |
| **Passing Events and Topics**... ... ... ... | iv |
| **Gifts and Bequests** ... ... ... ... ... | iv |
| **The Institutional Worker** ... .. (Suppl.) | 1 |
| The Ward Linen Supply: Answer to January Question. | |

